# 2-[Bis(2-amino­ethyl)amino]ethanaminium chloride dichloro­methane solvate

**DOI:** 10.1107/S1600536808039652

**Published:** 2008-11-29

**Authors:** Moritz M. Reichvilser, Tanja Ossiander, Peter Klüfers, Peter Mayer

**Affiliations:** a Ludwig-Maximilians-Universität, Department Chemie und Biochemie, Butenandtstrasse 5–13 (Haus D), 81377 München, Germany

## Abstract

In the title compound, C_6_H_19_N_4_^+^·Cl^−^·CH_2_Cl_2_, the non-H atoms of the ammonium ion show non-crystallographic *C*_3_ symmetry. The chloride ion is embedded in a framework of seven crystallographically independent hydrogen bonds (five N—H⋯Cl and two C—H⋯Cl), which form layers parallel to the (100) plane. Two N---H...N bonds also occur.

## Related literature

For the crystal structure of *N*,*N*,*N*-tris­(2-ammonio­ethyl)amine trichloride, see: Rasmussen & Hazell (1963 ▶); Hazell & Rasmussen (1968 ▶); Ilioudis *et al.* (2000 ▶). 
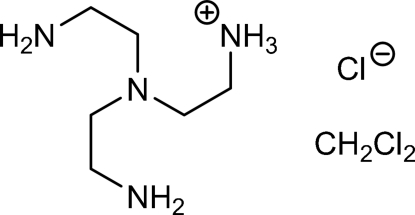


## Experimental

### 

#### Crystal data

C_6_H_19_N_4_^+^·Cl^−^·CH_2_Cl_2_
*M* *_r_* = 267.63
Monoclinic, 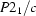

*a* = 12.1512 (4) Å
*b* = 8.5686 (2) Å
*c* = 13.5497 (3) Å
β = 104.273 (2)°
*V* = 1367.23 (6) Å^3^
*Z* = 4
Mo *K*α radiation
μ = 0.65 mm^−1^
*T* = 200 (2) K
0.17 × 0.17 × 0.17 mm

#### Data collection

Nonius KappaCCD area-detector diffractometer
Absorption correction: none
10639 measured reflections
3130 independent reflections
2431 reflections with *I* > 2σ(*I*)
*R*_int_ = 0.030

#### Refinement

*R*[*F* ^2^ > 2σ(*F* ^2^)] = 0.042
*wR*(*F* ^2^) = 0.111
*S* = 1.03
3130 reflections
127 parameters
H-atom parameters constrained
Δρ_max_ = 0.51 e Å^−3^
Δρ_min_ = −0.57 e Å^−3^

### 

Data collection: *COLLECT* (Hooft, 2004 ▶); cell refinement: *SCALEPACK* (Otwinowski & Minor, 1997 ▶); data reduction: *SCALEPACK* and *DENZO* (Otwinowski & Minor, 1997 ▶); program(s) used to solve structure: *SHELXS97* (Sheldrick, 2008 ▶); program(s) used to refine structure: *SHELXL97* (Sheldrick, 2008 ▶); molecular graphics: *ORTEP-3 for Windows* (Farrugia, 1997 ▶); software used to prepare material for publication: *SHELXL97*, *PLATON* (Spek, 200 ▶) and *Mercury* (Macrae *et al*., 2006 ▶).

## Supplementary Material

Crystal structure: contains datablocks global, I. DOI: 10.1107/S1600536808039652/zl2162sup1.cif

Structure factors: contains datablocks I. DOI: 10.1107/S1600536808039652/zl2162Isup2.hkl

Additional supplementary materials:  crystallographic information; 3D view; checkCIF report

## Figures and Tables

**Table 1:** Hydrogen-bond geometry (Å, °)

*D*—H⋯*A*	*D*—H	H⋯*A*	*D*⋯*A*	*D*—H⋯*A*
N2—H1⋯Cl1	0.94	2.54	3.3897 (17)	151
N2—H2⋯Cl1^i^	0.97	2.58	3.4402 (16)	148
N3—H3⋯Cl1^ii^	0.88	2.57	3.4013 (16)	159
N3—H4⋯Cl1	0.96	2.55	3.4203 (17)	150
N4—H5⋯Cl1	0.90	2.42	3.2562 (15)	153
N4—H6⋯N3^iii^	0.93	1.96	2.862 (2)	165
N4—H7⋯N2^iv^	1.02	1.73	2.746 (2)	171
C4—H14⋯Cl1^iii^	0.99	2.82	3.7014 (18)	149
C7—H21⋯Cl1^i^	0.99	2.54	3.482 (3)	160

Symmetry codes: (i) 
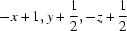
; (ii) 
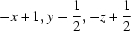
; (iii) 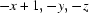; (iv) 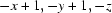.

**Fig. 1.**
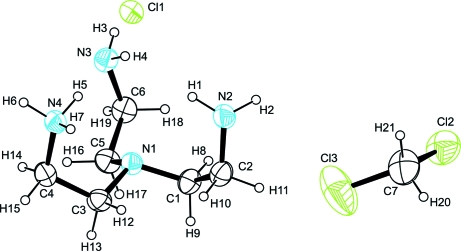
The molecular structure of the title compound, with atom labels and anisotropic displacement ellipsoids (drawn at 50% probability level) for non-H atoms.

**Fig. 2.**
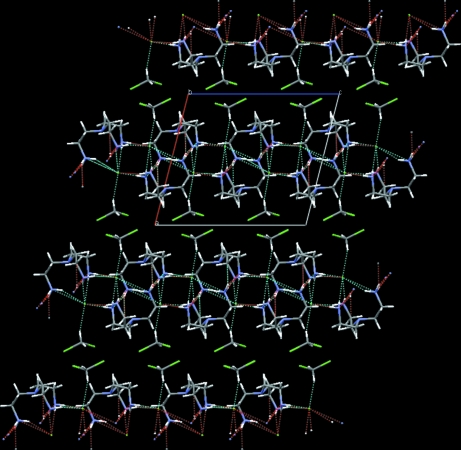
The packing and the hydrogen-bonded layers in the title compound, viewed along [0 1 0].

**Fig. 3.**
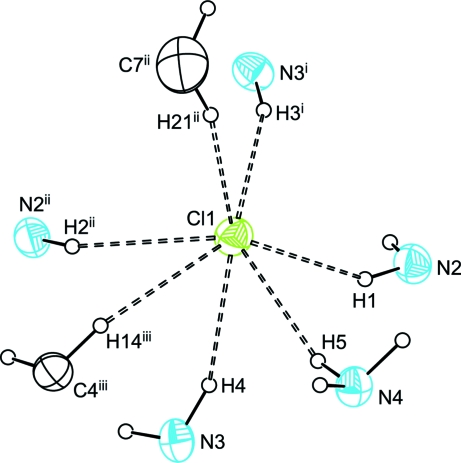
Hydrogen bonding to Cl1. [Symmetry codes: (i) 1 - x, 1/2 + y, 1/2 - z; (ii) 1 - x, -1/2 + y, 1/2 - z; (iii) 1 - x, -y, -z.]

## supplementary crystallographic information

### Comment

The title compounds molecular structure is shown in Fig. 1. The ammonium ion does not exhibit any crystallographic symmetry but, excluding the hydrogen atoms, it shows non-crystallographic *C*_3_ symmetry.

It has to be assumed that the chloride ion is formed by a nucleophilic substitution reaction between a part of the tris(2-aminoethyl)amine and the solvent dichloromethane.

In the crystal structure, hydrogen bonds between the ammonium and chloride ions and the solvate molecule form two-dimensional networks parallel to the (100) plane (see Fig. 2). The chloride ion is embedded in a framework of seven crystallographically independent hydrogen bonds (see Fig. 3).

### Experimental

Crystals of the title compound were obtained from a solution of tris(2-aminoethyl)amine (0.15 g, 5.0 mmol) and trimethylborate (0.52 g, 5.0 mmol) in dichloromethane (10 ml) upon slow evaporation of the solvent at room temperature.

### Refinement

All H atoms were found in difference maps. C-bonded H atoms were positioned geometrically (C—H = 0.99 Å) and treated as riding on their parent atoms [*U*_iso_(H) = 1.2*U*_eq_(C)]. N-bonded H atoms were assigned from difference maps and treated as riding on their parent atoms [*U*_iso_(H) = 1.2*U*_eq_(N)].

### Crystal data

**Table:**

C_6_H_19_N_4_^+^·Cl^–^·CH_2_Cl_2_	*F*_000_ = 568
*M**_r_* = 267.63	*D*_x_ = 1.300 Mg m^−^^3^
Monoclinic, *P*2_1_/*c*	Mo *K*α radiation λ = 0.71073 Å
Hall symbol: -P 2ybc	Cell parameters from 17190 reflections
*a* = 12.1512 (4) Å	θ = 3.1–27.5º
*b* = 8.5686 (2) Å	µ = 0.65 mm^−^^1^
*c* = 13.5497 (3) Å	*T* = 200 (2) K
β = 104.273 (2)º	Block, colourless
*V* = 1367.23 (6) Å^3^	0.17 × 0.17 × 0.17 mm
*Z* = 4	

### Data collection

**Table:**

Nonius KappaCCD area-detector diffractometer	3130 independent reflections
Radiation source: rotating anode	2431 reflections with *I* > 2σ(*I*)
Monochromator: MONTEL, graded multilayered X-ray optics	*R*_int_ = 0.030
Detector resolution: 9 pixels mm^-1^	θ_max_ = 27.5º
*T* = 200(2) K	θ_min_ = 3.2º
φ and ω scans	*h* = −15→15
Absorption correction: none	*k* = −11→10
10639 measured reflections	*l* = −17→17

### Refinement

**Table:**

Refinement on *F*^2^	Secondary atom site location: difference Fourier map
Least-squares matrix: full	Hydrogen site location: difference Fourier map
*R*[*F*^2^ > 2σ(*F*^2^)] = 0.042	H-atom parameters constrained
*wR*(*F*^2^) = 0.111	*w* = 1/[σ^2^(*F*_o_^2^) + (0.0471*P*)^2^ + 0.7878*P*] where *P* = (*F*_o_^2^ + 2*F*_c_^2^)/3
*S* = 1.03	(Δ/σ)_max_ < 0.001
3130 reflections	Δρ_max_ = 0.51 e Å^−^^3^
127 parameters	Δρ_min_ = −0.57 e Å^−^^3^
Primary atom site location: structure-invariant direct methods	Extinction correction: none

### Special details

**Table:**

Geometry. All e.s.d.'s (except the e.s.d. in the dihedral angle between two l.s. planes) are estimated using the full covariance matrix. The cell e.s.d.'s are taken into account individually in the estimation of e.s.d.'s in distances, angles and torsion angles; correlations between e.s.d.'s in cell parameters are only used when they are defined by crystal symmetry. An approximate (isotropic) treatment of cell e.s.d.'s is used for estimating e.s.d.'s involving l.s. planes.
Refinement. Refinement of *F*^2^ against all reflections. The weighted *R*-factor *wR* and goodness of fit *S* are based on *F*^2^, conventional *R*-factors *R* are based on *F*, with *F* set to zero for negative *F*^2^. The threshold expression of *F*^2^ > 2*σ*(*F*^2^) is used only for calculating *R*-factors(gt) *etc*. and is not relevant to the choice of reflections for refinement. *R*-factors based on *F*^2^ are statistically about twice as large as those based on *F*, and *R*-factors based on all data will be even larger.

### Fractional atomic coordinates and isotropic or equivalent isotropic displacement parameters (Å^2^)

**Table:**

	*x*	*y*	*z*	*U*_iso_*/*U*_eq_	
N1	0.28042 (13)	0.26045 (16)	−0.00985 (11)	0.0291 (3)	
N2	0.40189 (14)	0.52948 (18)	0.11822 (12)	0.0355 (4)	
H1	0.4350	0.4312	0.1169	0.043*	
H2	0.3888	0.5495	0.1846	0.043*	
N3	0.36750 (14)	0.01291 (18)	0.14123 (12)	0.0347 (4)	
H3	0.3757	−0.0326	0.2006	0.042*	
H4	0.4111	0.1080	0.1487	0.042*	
N4	0.48946 (13)	0.20788 (18)	−0.07457 (11)	0.0324 (3)	
H5	0.4973	0.2055	−0.0066	0.039*	
H6	0.5435	0.1373	−0.0843	0.039*	
H7	0.5240	0.3119	−0.0890	0.039*	
C1	0.22269 (16)	0.3910 (2)	0.02691 (15)	0.0362 (4)	
H8	0.2052	0.3602	0.0918	0.043*	
H9	0.1497	0.4113	−0.0232	0.043*	
C2	0.29173 (17)	0.5407 (2)	0.04382 (15)	0.0364 (4)	
H10	0.3049	0.5746	−0.0222	0.044*	
H11	0.2462	0.6228	0.0666	0.044*	
C3	0.28825 (16)	0.2874 (2)	−0.11498 (13)	0.0342 (4)	
H12	0.3085	0.3979	−0.1226	0.041*	
H13	0.2132	0.2678	−0.1621	0.041*	
C4	0.37594 (16)	0.1831 (2)	−0.14384 (13)	0.0343 (4)	
H14	0.3534	0.0725	−0.1405	0.041*	
H15	0.3792	0.2058	−0.2147	0.041*	
C5	0.22065 (16)	0.1131 (2)	−0.00284 (14)	0.0355 (4)	
H16	0.2424	0.0354	−0.0487	0.043*	
H17	0.1378	0.1309	−0.0268	0.043*	
C6	0.24633 (17)	0.0466 (2)	0.10427 (14)	0.0362 (4)	
H18	0.2230	0.1224	0.1504	0.043*	
H19	0.2022	−0.0505	0.1044	0.043*	
Cl2	0.03789 (6)	0.73386 (9)	0.38860 (6)	0.0703 (2)	
Cl3	0.07369 (8)	0.62211 (16)	0.19725 (8)	0.1163 (4)	
C7	0.1040 (2)	0.7685 (3)	0.2895 (2)	0.0630 (7)	
H20	0.0780	0.8702	0.2574	0.076*	
H21	0.1871	0.7746	0.3178	0.076*	
Cl1	0.60198 (4)	0.24526 (5)	0.16855 (3)	0.03439 (14)	

### Atomic displacement parameters (Å^2^)

**Table:**

	*U*^11^	*U*^22^	*U*^33^	*U*^12^	*U*^13^	*U*^23^
N1	0.0323 (8)	0.0290 (7)	0.0272 (7)	−0.0003 (6)	0.0093 (6)	0.0019 (6)
N2	0.0442 (9)	0.0298 (8)	0.0320 (8)	0.0009 (7)	0.0082 (7)	−0.0019 (6)
N3	0.0409 (9)	0.0321 (8)	0.0333 (8)	0.0032 (7)	0.0132 (7)	0.0056 (6)
N4	0.0375 (8)	0.0331 (8)	0.0276 (7)	0.0026 (7)	0.0101 (6)	0.0001 (6)
C1	0.0329 (10)	0.0361 (10)	0.0412 (10)	0.0026 (8)	0.0123 (8)	−0.0008 (8)
C2	0.0398 (10)	0.0306 (9)	0.0403 (10)	0.0042 (8)	0.0127 (8)	0.0019 (8)
C3	0.0372 (10)	0.0371 (10)	0.0285 (9)	0.0024 (8)	0.0082 (8)	0.0051 (7)
C4	0.0394 (10)	0.0344 (10)	0.0296 (9)	−0.0016 (8)	0.0095 (8)	−0.0031 (7)
C5	0.0350 (10)	0.0347 (10)	0.0355 (9)	−0.0069 (8)	0.0065 (8)	0.0000 (8)
C6	0.0383 (10)	0.0352 (10)	0.0382 (10)	−0.0026 (8)	0.0151 (8)	0.0037 (8)
Cl2	0.0586 (4)	0.0835 (5)	0.0745 (4)	0.0079 (3)	0.0269 (3)	0.0044 (3)
Cl3	0.0780 (6)	0.1752 (11)	0.1012 (7)	−0.0436 (6)	0.0326 (5)	−0.0730 (7)
C7	0.0437 (13)	0.0711 (17)	0.0787 (18)	−0.0073 (12)	0.0239 (13)	−0.0116 (14)
Cl1	0.0357 (3)	0.0366 (3)	0.0302 (2)	−0.00045 (18)	0.00691 (17)	−0.00011 (17)

### Geometric parameters (Å, °)

**Table:**

N1—C3	1.469 (2)	C2—H10	0.9900
N1—C1	1.471 (2)	C2—H11	0.9900
N1—C5	1.471 (2)	C3—C4	1.514 (3)
N2—C2	1.467 (2)	C3—H12	0.9900
N2—H1	0.9358	C3—H13	0.9900
N2—H2	0.9660	C4—H14	0.9900
N3—C6	1.462 (2)	C4—H15	0.9900
N3—H3	0.8776	C5—C6	1.518 (3)
N3—H4	0.9638	C5—H16	0.9900
N4—C4	1.480 (2)	C5—H17	0.9900
N4—H5	0.9019	C6—H18	0.9900
N4—H6	0.9265	C6—H19	0.9900
N4—H7	1.0244	Cl2—C7	1.751 (3)
C1—C2	1.519 (3)	Cl3—C7	1.745 (3)
C1—H8	0.9900	C7—H20	0.9900
C1—H9	0.9900	C7—H21	0.9900
			
C3—N1—C1	111.05 (14)	C4—C3—H12	109.2
C3—N1—C5	110.38 (14)	N1—C3—H13	109.2
C1—N1—C5	110.28 (14)	C4—C3—H13	109.2
C2—N2—H1	111.7	H12—C3—H13	107.9
C2—N2—H2	107.2	N4—C4—C3	110.89 (15)
H1—N2—H2	110.4	N4—C4—H14	109.5
C6—N3—H3	106.2	C3—C4—H14	109.5
C6—N3—H4	110.5	N4—C4—H15	109.5
H3—N3—H4	110.1	C3—C4—H15	109.5
C4—N4—H5	119.5	H14—C4—H15	108.0
C4—N4—H6	113.5	N1—C5—C6	113.30 (15)
H5—N4—H6	103.3	N1—C5—H16	108.9
C4—N4—H7	111.5	C6—C5—H16	108.9
H5—N4—H7	105.6	N1—C5—H17	108.9
H6—N4—H7	101.7	C6—C5—H17	108.9
N1—C1—C2	113.68 (15)	H16—C5—H17	107.7
N1—C1—H8	108.8	N3—C6—C5	110.71 (15)
C2—C1—H8	108.8	N3—C6—H18	109.5
N1—C1—H9	108.8	C5—C6—H18	109.5
C2—C1—H9	108.8	N3—C6—H19	109.5
H8—C1—H9	107.7	C5—C6—H19	109.5
N2—C2—C1	115.12 (15)	H18—C6—H19	108.1
N2—C2—H10	108.5	Cl3—C7—Cl2	111.83 (15)
C1—C2—H10	108.5	Cl3—C7—H20	109.2
N2—C2—H11	108.5	Cl2—C7—H20	109.2
C1—C2—H11	108.5	Cl3—C7—H21	109.2
H10—C2—H11	107.5	Cl2—C7—H21	109.2
N1—C3—C4	112.06 (15)	H20—C7—H21	107.9
N1—C3—H12	109.2		
			
C3—N1—C1—C2	−69.7 (2)	N1—C3—C4—N4	−58.4 (2)
C5—N1—C1—C2	167.57 (15)	C3—N1—C5—C6	158.47 (16)
N1—C1—C2—N2	−59.7 (2)	C1—N1—C5—C6	−78.5 (2)
C1—N1—C3—C4	162.77 (15)	N1—C5—C6—N3	−60.7 (2)
C5—N1—C3—C4	−74.60 (19)		

### Hydrogen-bond geometry (Å, °)

**Table:**

*D*—H···*A*	*D*—H	H···*A*	*D*···*A*	*D*—H···*A*
N2—H1···Cl1	0.94	2.54	3.3897 (17)	151
N2—H2···Cl1^i^	0.97	2.58	3.4402 (16)	148
N3—H3···Cl1^ii^	0.88	2.57	3.4013 (16)	159
N3—H4···Cl1	0.96	2.55	3.4203 (17)	150
N4—H5···Cl1	0.90	2.42	3.2562 (15)	153
N4—H6···N3^iii^	0.93	1.96	2.862 (2)	165
N4—H7···N2^iv^	1.02	1.73	2.746 (2)	171
C4—H14···Cl1^iii^	0.99	2.82	3.7014 (18)	149
C7—H21···Cl1^i^	0.99	2.54	3.482 (3)	160

Symmetry codes: (i) −*x*+1, *y*+1/2, −*z*+1/2; (ii) −*x*+1, *y*−1/2, −*z*+1/2; (iii) −*x*+1, −*y*, −*z*; (iv) −*x*+1, −*y*+1, −*z*.
